# From urban planning and emergency training to Pokémon Go: applications of virtual reality GIS (VRGIS) and augmented reality GIS (ARGIS) in personal, public and environmental health

**DOI:** 10.1186/s12942-017-0081-0

**Published:** 2017-02-20

**Authors:** Maged N. Kamel Boulos, Zhihan Lu, Paul Guerrero, Charlene Jennett, Anthony Steed

**Affiliations:** 1 The Alexander Graham Bell Centre for Digital Health, Moray College UHI, University of the Highlands and Islands, Moray Street, Elgin, IV30 1JJ Scotland, UK; 2 Department of Computer Science, University College London, 66-72 Gower Street, London, WC1E 6EA England, UK

**Keywords:** VRGIS (virtual reality GIS), ARGIS (augmented reality GIS), Urban planning, Environmental planning, Smart cities, Emergency training, Physical activity, Public health, Pokémon Go

## Abstract

The latest generation of virtual and mixed reality hardware has rekindled interest in virtual reality GIS (VRGIS) and augmented reality GIS (ARGIS) applications in health, and opened up new and exciting opportunities and possibilities for using these technologies in the personal and public health arenas. From smart urban planning and emergency training to Pokémon Go, this article offers a snapshot of some of the most remarkable VRGIS and ARGIS solutions for tackling public and environmental health problems, and bringing about safer and healthier living options to individuals and communities. The article also covers the main technical foundations and issues underpinning these solutions.

## Background

Virtual reality GIS (VRGIS) is decades old. It has been around since the 1990s [1, 2]. (One may also add mixed reality GIS [MRGIS] and augmented reality GIS [ARGIS] as closely related terms and concepts.) But more recent developments in technologies, such as big data, augmented reality, graphic processing units (GPUs) and the Internet of Things (IoT), have helped provide superior implementations, higher performance and better human–computer interactive modes for VRGIS, which enabled its use in solving more complex, practical and real-world problems.

VRGIS technology is a combination of virtual reality (VR) and GIS technologies, integrating three-dimensional GIS (3D GIS) and Internet-oriented GIS (Web GIS). VRGIS technology adopts different human–computer interaction devices [1]. It establishes a three-dimensional (3D) model in a virtual environment, and operates via personal computers, mobile devices and smart glasses. Newer generations of low-cost hardware technologies and ubiquitous devices are significantly reducing the threshold of VRGIS adoption and acceptance by various research communities and user groups [3].

**Fig. 1:**
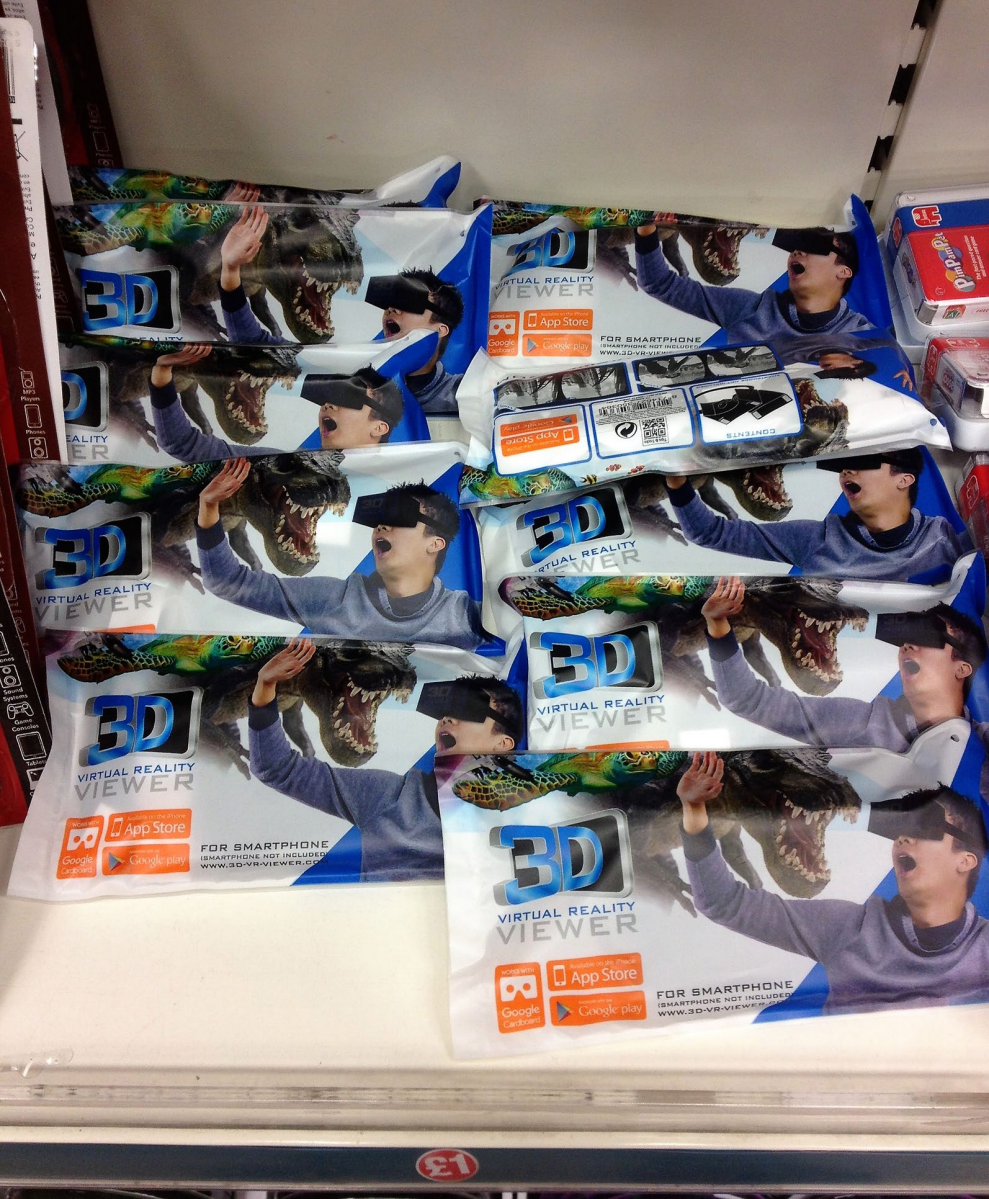
Due to their very low manufacturing costs, it is not uncommon these days to find Google Cardboard-based VR sets, such as the ones shown in this photo, offered for prices as low as GBP £1 or EUR €1.5 in variety stores across the UK and Europe, or even free of charge from many sources as promotional gift items

Indeed, the year 2016 brought new opportunities for more mature and accessible mobile and non-mobile VRGIS and ARGIS applications, with the release of mainstream immersive VR hardware gear by major players, spanning a wide range of prices, levels of sophistication and functionalities, such as Google (Daydream View VR [4] and its much cheaper predecessor, Cardboard, introduced in 2014 [5], which uses the smartphone’s gyroscope for head tracking—Fig. 1), Microsoft (HoloLens [6] and cheaper VR headsets [7]), Valve and HTC (Vive [8]), Facebook and Samsung (Oculus Rift and Gear VR [9]), and Sony (PlayStation VR [10]).

## A quick overview of recent VRGIS and ARGIS/MRGIS applications in health and public health

### Smart healthy cities (urban and environmental planning)

In the realm of smart healthy cities [11, 12], VRGIS offers unique simulation and visualisation opportunities for use in urban and environmental planning, as well as in impact assessment. Esri CityEngine, which is used in urban planning and design [13, 14], has been successfully coupled with Oculus Rift to produce a powerful VRGIS solution for more participatory, smarter urban planning [15, 16]. Thanks to its powerful immersive visualisation approach, the platform can be used to better engage with, and collect the opinion of, stakeholders and citizens/communities about any proposed future city plans affecting the places they live and work in. For example, neighbourhood walkability can be tested (bike paths, walking paths, etc.), and levels of city and street noise at various times of the day can be simulated (if combined with a 3D spatial audio solution and appropriate models of city noise sources and levels). Readers wishing to get some rough idea of what an Esri CityEngine with Oculus Rift experience is like (without the price tag attached to it) can try out Google Street View under Cardboard [17]. A closer look at ‘VRGIS for smart cities’ follows later in this article.

### Mass casualty education and emergency training

VRGIS can also be used for mass casualty education and in military and emergency training, as part of a city or country’s emergency preparedness provisions. Evacuation and response scenarios in natural and man-made disasters can be simulated (planned and rehearsed) in an immersive 3D VR environment using GIS data and real city models. Realistic and immersive virtual accident scenes can be offered for trainees to interact with at a fraction of the cost of a comparable large-scale conventional drill in the physical (real) world [18–20].

### Personal health and well-being (promotion of physical activity and reduction of stress)

**Fig. 2:**
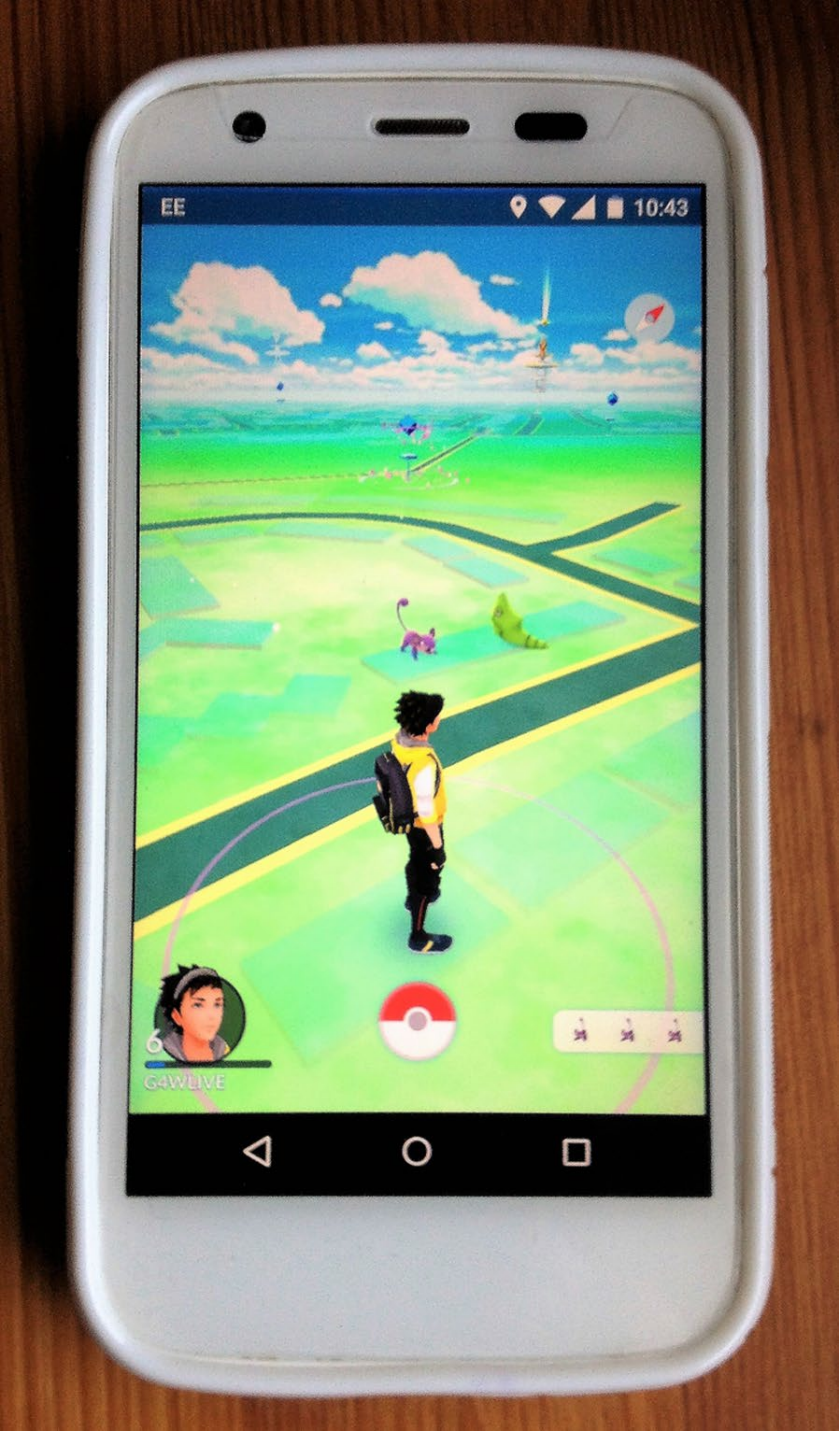
Pokémon Go running on an Android smartphone. Note the cartoon-style map of the user’s real world location. Nearby Pokémon creatures, PokeStops (where the player can collect in-game items, such as Eggs and Poké Balls [used to catch Pokémon creatures]) and Pokémon Go Gyms (battle arenas of the Pokémon world) are overlaid on the map. Players are expected to walk or run towards these creatures and points of interest to interact with them, while competing with one another and in teams. (All players at a given location and time see the same creatures and points of interest around them.) In AR (Augmented Reality) mode, the game uses the smartphone’s camera to overlay Pokémon creatures (as they are being caught by the player) onto real-world scenery

Physical activity (in the right amount) is a key ingredient of a healthy lifestyle. It improves physical and psychological well-being, reduces stress, decreases a person’s risk of developing major diseases, such as type 2 diabetes, heart disease, stroke and cancer, and lowers the risk of pre-mature death. Introduced in July 2016, Pokémon Go [21] (Fig. 2), a mobile location-based social exergame [22] with potential and documented health benefits (e.g., [23–25]), is perhaps the most popular ever example of a health-related ARGIS application. Created by the same experts behind Google Earth [26], Pokémon Go relies heavily on the location services of smartphones (GPS [Global Positioning System], Wi-Fi, and mobile networks) to deliver its multiplayer experience, but not without documented and potential misuses or safety and privacy concerns [27–30].

Like very many other games, Pokémon Go’s somewhat fading user interest and declining user base was to be expected, particularly when accounting for the initial media hype that drove many people to the game only temporarily, to satisfy their curiosity [31]. Nevertheless, updates to Pokémon Go [32] and merchandise for further monetisation of the game, such as the Pokémon Go Plus wearable [33], are still being released as of January 2017. But the successive updates of the game have also made it increasingly “heavier” and bloated, and consequently slower or totally unplayable on less powerful, low-end devices. For example, while the Apple iPhone 4S was able to run the earliest versions of Pokémon Go almost flawlessly, newer versions of the game (as of January 2017) are unplayable on that handset, crashing with each attempt to run the game. Areas for improvement in future editions of Pokémon Go and similar games include ‘game loading times’ (need to be faster) and ‘battery drain’ (needs to be reduced).

Furthermore, games are in some ways like fashion, and many people are now looking forward to the next geosocial game to follow after Pokémon Go. Most people will get bored of a game at some point if not after a couple of months then after 4 or 6 months. Furthermore, Pokémon Go has a finite number of levels after which the player would be considered to have completed it. Most players will never reach beyond a certain level. They will give up, either as the game gets much harder or as its monetisation kicks in (when progression becomes strictly tied to in-game purchases or extremely slow without them to force players to buy something with real currency). But to play an exergame for even such a limited duration of a few or several months is still beneficial, worthy and commendable (though some Pokémon Go players were cheating to progress in-game instead of doing real physical activity [34]—tying future exergames to heart rate and other relevant body sensors can help minimise this kind of cheating and provide better feedback to players).

Building on his earlier concept of a Kinect-based NUI (natural user interface) for 3-D virtual globe navigation [35], Kamel Boulos (first author on this article) is further proposing a stationary bike or treadmill coupled with an immersive VR (Cardboard or better) version of Google Street View/Google Earth, whereby the player’s steps are translated to (or rewarded by) entertaining and culturally rich virtual promenades along the Champs-Élysées in Paris, France (for example), and other interesting landmarks around the world. The changing scenery (virtual tourism) would serve as a stimulus or motive for the player to continue exercising on the stationary bike or treadmill without getting bored or giving up the activity too soon. A social component (over the Internet) can be added, whereby other players (friends and family members running or biking on similar stationary devices in the same building or elsewhere) can be “projected” into the same immersive space, and communication via voice chat enabled, so that the player is not alone and is joined by other players during these virtual promenades (another motive for the player to continue exercising). Spatialised 3D audio of ambient sounds can further bring the streets, cities and countryside in the immersive virtual environment to life. This type of VRGIS involving physical activity would not just benefit a person’s physical health, but also the player’s mental health, helping the person better manage her/his stress levels. Virtual sunshine in such immersive environments might also exert some additional mood lifting effects [36], but research is needed to establish whether or not the same sunlight-induced effects on serotonin and melatonin regulation apply in immersive VR. A 15-min break involving the above setup during a busy and stressful office day can prove helpful and beneficial to employees’ health, particularly in those circumstances in which there is no convenient access to suitable nearby outdoor recreational places for walking, jogging, or cycling (e.g., during those dark, cloudy and rainy winter days).

### Healthier and safer living (car accident prevention, healthy food outlet suggestions, etc.)

Another noteworthy ARGIS application that can help improve road safety and prevent car accidents (think ‘smart cars’, a component of smart healthy cities) is iOnRoad, available on Android and iOS [37, 38]. One can also think of a useful ARGIS app that augments nearby restaurants located on a map or camera photo with healthy (and non-healthy) eating information, and can also tailor recommendations based on user’s health condition, e.g., offer special restaurant recommendations for people with diabetes (cf. the Layar layer for recommending top-rated, nearby Asian restaurants in and round Los Angeles, USA, demonstrated in [39]).

The remainder of this article covers the main technical foundations and issues underpinning VRGIS solutions today, with some emphasis on VRGIS-based application tools for smart cities.

## Characteristics of VRGIS

Online virtual worlds and 3D stereoscopic solutions (considered as foundation technologies for VRGIS and ARGIS) have a great potential for being used for research purposes in social, behavioural, economic and human-centred computer sciences, with many applications in the public and environmental health arenas [40–44]. In geography, including human and health geography, virtual worlds have been successfully developed as assistant tools, facilitating the creation of studies and the understanding of the theories and practices of geography [45–49].

VRGIS can be seen as an enhanced version of geographical virtual worlds. VRGIS, which merges 3D stereoscopic, VR and GIS technologies, uses footprint files in GIS format for 3D reconstruction [50], and expresses GIS information in the VR domain based on a coupled system; the VRGIS method consists of GIS and VR modules [51]. When operating VRGIS in the virtual environment, users can interact with the system and get feedback from it using different sensing devices. The external world and the system can form a feedback loop through sensing devices.

The main characteristics of VR technology are (1) interactivity—the extent to which the users can operate and get feedback in the simulated environment; (2) existence—the extent of the user’s presence in the simulation environment; (3) autonomy—the movement degree of objects in the virtual environment based on physical laws; and (4) multiple perceptibility [1]—compared with traditional GIS, VRGIS’ main strength lies in the enhanced interaction between the users and the system, which improves the user’s experience making it more immersive.

Thanks to modern multimedia, mass storage technologies and linkages through broadband networks, VRGIS is able to combine remote sensing (RS), aerial photogrammetry, GPS, GIS, city simulation, virtual displays and other technologies to conduct detailed 3D descriptions of a multi-resolution, multi-scale complex geographical environment, with multiple spatio-temporal categories. This is where past, present, and future geographical environments are rendered in a realistic and immersive manner with digital virtual reality via computer networks and other information technologies [51, 52].

## Key issues of present-day VRGIS

### Modelling technology in the dynamic environment

The virtual worlds that are explored in VRGIS range from natural landscapes to urban cityscapes. Many VRGIS applications require the worlds to have a certain amount of detail in order to be useful. City planners, for example, need to identify the exact 3D shape of each building to check if several regulations, such as protected views in a city, are met. Energy companies responsible for planning solar installations for greener and more sustainable environments may be interested in the size and slant of city roofs, including the occlusion of roofs by nearby buildings. To meet these requirements, the 3D models that represent these environments should be large and detailed, which makes them extremely complex.

Traditionally, there are two main technical challenges in handling this complexity. Firstly, as VR is required to be interactive, the models should be displayed in real-time with over 30 frames per second (fps) and ideally at about 60 fps to reduce the input lag experienced by users [53, 54]. Rendering technology has matured over the past decades to a point where a non-photorealistic display of these complex models at the interactive frame rates is achievable, mainly by organising the models into spatial hierarchies [55, 56], as implemented, for example, in Cesium 3D Tiles [57], and by decreasing the level of detail in the distant geometry [58]. Web-based technologies for rendering [59, 60] and 3D modelling [61–63] have been on the rise recently, and some of them are specifically designed for 3D GIS visualisations [57].

Secondly, it is difficult to create the large amounts of geometric data that are required for these models in a reasonable amount of time. Especially for detailed urban environments, this poses a great challenge that has gained much attention in recent computer graphics research [64]. Data can be obtained from satellite, aerial or street-level images using photogrammetry [65–68], range scanners such as LIDAR (Light Detection and Ranging) [69, 70], or through manual modelling to either improve existing data [71, 72] or to create data from scratch [73, 74], possibly for a planned project where no real-world objects are available. These approaches vary in the fidelity of the generated data, the required amount of user interaction and the availability of higher-level information in the reconstructed models. High-level information may, for example, be semantic labels for objects such as roofs and doors that may be useful Building Information Modelling (BIM), or information that may help to modify buildings efficiently, such as a procedural model of a building or other semantically meaningful parameterisations. Typically, approaches that require little user interaction tend to have lower-fidelity results, with less high-level information. The most prominent example of an approach that requires little user interaction is probably Google Earth [75], which discontinued manual building modelling with Google Building Maker [76] in favour of a more automatic photogrammetric reconstruction. The methods that require more user interaction lie on the other side of the spectrum, such as procedural modelling programs, e.g., CityEngine [13, 77], and traditional polygon modelling programs, e.g., Google SketchUp [78], AutoCAD [79] and OnShape [61]. Examples of companies that do urban reconstruction with more manual modelling than Google Earth are Esri [80] and VertexModeling [81].

More recent developments have focused on facilitating manual modelling of complex geometry by making use of higher-level model structure [73, 74, 82, 83], or introducing manual guidance into automatic reconstruction to achieve higher-quality results [71, 72, 84]. These approaches promise a more favourable trade-off between the required amount of user interaction and the resulting model quality, but have not yet established themselves in the industry.

### Crowdsourcing

‘Wikification of GIS by the masses’ (WGM) [85], also known as Volunteered Geographic Information (VGI) [86], is the specific embodiment of crowdsourcing approach in GIS. Following the emergence of the Social Web (or Web 2.0) and improvements in mobile devices, the growth of spatial data is no longer limited to activities carried out by specialist organisations. A paradigm shift emerged, whereby individuals and informal institutions can now collect and disseminate their own geographical knowledge. Users contribute to mapping projects for a variety of reasons, including learning about, and sharing, local knowledge, supporting the general principle of the free availability of mapping data, and for the attached importance of, and recognition by, the community they serve [87]. Official ‘mapping party’ events are also organised in different locations around the world, inviting local users to socialise and engage with newcomers as they map [88].

User-generated maps from projects such as OpenStreetMap (OSM) have been recognised for their impressive levels of detail [89], and have emerged as an important source of information for supporting disaster management [90], among other applications. However, this progress in user-generated maps has largely been limited to 2D (two-dimensional) maps, though some user-generated 3D models are available on Google Earth [91]. Researchers are currently exploring the potential of extending user-generated maps to the next dimension, that of 3D mapping [50, 92–94], and new tools are being created to support the generation and consumption of VR maps, e.g., Google Earth VR [95].

Collaborative mapping in 3D is more difficult than in 2D, because it requires users to have basic knowledge and skills of 3D modelling. Also WGM data are heterogeneous in quality, completeness and accuracy, which makes 3D reconstruction difficult [93]. Yet, if these challenges can be overcome, the idea of a comprehensive user-generated 3D map of the world presents many exciting possibilities. For example, a 3D model of the city of London could be a shared resource for planning, tourism and heritage, or an extremely large user-created fantasy virtual world that can underpin the next generation of massive-multiplayer games [50].

### System integration and interaction technology

GIS is not only a system, but also science [96, 97]. The geographic information provides multi-source, multi-dimensional, multi-scale, multi-spatio-temporal model and perceptual details. Data fusion and integration technologies are of great importance to the system. GIS data share some ‘big data’ characteristics, such as being large scale, diverse, of varying predictability and often available in real-time [98]. With regard to big data, the system integration technology mainly involves the integration of spatial data, storage management model and synchronisation technology [99], and user space positioning technology and human data [100]. These technologies can be summarised as network and communication (NC) technology and human–computer interaction (HCI) technology. NC focuses on system integration, while HCI focuses on system interaction, with both technology classes bridging the gap between users and VRGIS.

### Network and communication technology

Traditional distributed VRGIS uses the network to connect users in the virtual environment and carry out distributed data sharing and multi-user real-time interaction [101, 102]. The Internet of Things [12] utilises a new generation of sensors to link the real objects in the physical world with the Internet according to set agreements involving information exchange and communication, so as to achieve intelligent identification, positioning, tracking, monitoring and resource management. This leads to the conversion of VRGIS from ‘digitisation’ to ‘intelligence’. Meanwhile, cloud computing technology provides a more powerful computing power for the analysis, prediction, abstraction and visualisation of geographic data.

### Human–computer interaction interface

Spatial representations have gone beyond the visual [103]. The earliest VR used Cave Automatic Virtual Environment (CAVE) and other large-scale 3D display technology along with 3D glasses [104] to provide users with a sense of immersion [105]. Traditional interaction techniques, such as data gloves, have their disadvantages and can be inconvenient to use. They have low resolution, small scope of action, and can cause serious delay. The latest interactive devices and technologies improved the tracking range and accuracy of the virtual reality [106, 107] by simplifying the 3D display device structure and reducing cost. Also, the enhancements provided better quality and superior visual effect of the 3D image [108, 109], along with improving user’s experience. For instance, Oculus Rift can be connected to PC (personal computer) games, providing convenient visual immersion for players. Augmented and mixed reality technologies and devices such as Google Glass (discontinued in 2015) [110] and Microsoft HoloLens [6] facilitate users’ interaction with real (physical) and virtual worlds, seamlessly merging and integrating the two realms.

## VRGIS-based application tools for smart cities

Many users are satisfied with the realistic visual effects and immersive interactive experience of the latest VRGIS developments. The key to a successful VRGIS application lies in identifying suitable application areas [1], and fully recognising the efficacy of VRGIS and the convenience it can provide to various fields [111]. The design of well-tailored application tools requires not only familiarity with the feasibility of VRGIS implementation in the scenarios at hand, but also an in-depth understanding of the corresponding industrial demands. VRGIS experts carry out demand analyses and functional design of application tools to achieve the desired practical goals in each case, effectively improving labour efficiencies in the corresponding application domains [48, 112]. After the system implementation of VRGIS in a given field, effectiveness can be measured using appropriate human–computer interaction and usability testing methods to study users [113]. Final judgments about the application are then made based on users’ feedback results [114, 115].

VRGIS has been widely applied in domains related to smart cities and urban management, such as traffic and transportation [116], e-government and e-business [98], ocean and island management [117–119], scenic area management [120], virtual community [121], geography education [122, 123], and underground pipeline management [124]. Virtual geographies are currently being developed for several applications, including virtual cities, landscape visualisation, visualising past and future geographies, visualising abstract concepts and taking people on virtual field courses [125–127].

Traditional urban and environmental applications of VRGIS focus on the simulation of landscape structure [120]; geomorphological structure; geological structure; large-scale traffic and urban engineering structure; natural movements of the Earth system, such as volcanic eruptions, earthquake processes, flood disasters and other emergency cases; desertification and soil erosion processes in lakes, forests and other natural ecosystems; regional, urban and other ecological systems [128]; sustainable development of cities [129]; and transformation and planning of regions. In the context of smart cities, VRGIS has been applied to population management, traffic (congestion) prediction and mitigation, urban resource allocation, monitoring of water resources, environmental protection, disaster prevention, early warning systems and rescue planning operations.

In a modern smart city system, the most basic characteristic of VRGIS is its capacity to visualise 3D details. Users immersed in the virtual environment can test different possibilities and candidate locations for a given task or a new city development plan to decide on the best course of action to take [130, 131]. Planners of new buildings or other facilities can have a comprehensive view of their new development location from various perspectives, including surrounding and nearby buildings. Users, such as city managers, can see the actual landscape of streets, buildings and vehicles, and assess the number of buildings, congestion conditions and light exposure within the vicinity.

The core function of geographic information technology as decision support tool plays a key role in realising the full potential of smart cities and associated technologies, such as IoT [100, 131, 132]. Capitalising on the technology’s powerful spatio-temporal decision support affordances, users can interrogate the relevant GIS databases to analyse and display the distribution of business activities of interest, such as the positioning of public facilities, major pipelines and other useful information. An emergency alarm service enables the officers in charge to immediately obtain 3D images of areas requiring immediate attention for further processing. By properly integrating event information in the GIS database, city officers can see, and dynamically monitor, the location(s) of corresponding smart city object(s) attached to an event as it unfolds, such as specific building(s) or particular street(s) or crossing(s) in the case of a traffic jam.

## Conclusions

The rapid developments in computer technology are opening up new application frontiers for VRGIS and ARGIS in the domains of personal, public and environmental health. When properly conceived and implemented, VRGIS and related technologies can serve as enablers of healthier and safer living for individuals and communities. IoT-driven smart cities benefit the most from the real-time, effective and highly integrated 3D visualisation of big (geo)data via VRGIS. VRGIS can assist in the analysis of the urban fabric and other related attributes, providing auxiliary methods for urban and environmental planning and design [133, 134]. The underpinning databases can be updated with 3D data at any time (even in real time), so as to reflect new urban plans and regional changes in a timely manner (or as they unfold in real time) via VRGIS, thus broadening the technology’s application prospects.

Looking at the future, there are virtually no limits to the possibilities of VRGIS and ARGIS applications in health and medicine. For example, GIS has traditionally been applied to features on the surface of the Earth, but it is also possible to apply the technology to anatomical features at the scale of the human body [135]. In this respect, one may consider Royal Philips’ augmented-reality surgical navigation technology for image-guided spine, cranial and trauma surgery [136, 137] as an advanced and highly specialised form of ARGIS/MRGIS. News updates related to the topic of this article can be always accessed at [138].
